# Surface-Treated versus Untreated Large-Bore Catheters as Vascular Access in Hemodialysis and Apheresis Treatments

**DOI:** 10.1155/2012/956136

**Published:** 2012-04-10

**Authors:** Rolf Bambauer, Ralf Schiel, Carolin Bambauer, Reinhard Latza

**Affiliations:** ^1^Institute for Blood Purification, Frankenstraße 4, 66424 Homburg, Germany; ^2^Inselklinik Heringsdorf GmbH, 17424 Seeheilbad Heringsdorf, Germany; ^3^Main Hospital Darmstadt, 64283 Darmstadt, Germany; ^4^Laboratorium of Medicine, 66386 St Ingbert, Germany

## Abstract

*Background*. Catheter-related infections, thrombosis, and stenosis are among the most frequent complications associated with catheters, which are inserted in vessels. Surface treatment processes of the outer surface, such as ion-beam-assisted deposition, can be used to mitigate such complications. *Methods*. This retrospective study (1992–2007) evaluated silver-coated (54 patients) and noncoated (105 patients) implanted large-bore catheters used for extracorporeal detoxification. The catheters were inserted into the internal jugular or subclavian veins. After removal, the catheters were cultured for bacterial colonization using standard microbiologic assays. They also were examined using scanning electron microscope. *Results*. The silver coated catheters showed a tendency towards longer in situ time. The microbiologic examinations of the catheter tips were in both catheter types high positive, but not significant. *Conclusion*. The silver-coated catheters showed no significantly reduction in infection rate by evaluation of all collected data in this retrospective study. There was no association between both catheters in significantly reducing savings in treatment costs and in reducing patient discomfort. Other new developed catheter materials such as the microdomain-structured inner and outer surface are considered more biocompatible because they mimic the structure of natural biological surface.

## 1. Introduction

Since the introduction of large-bore catheters for acute hemodialysis more than 50 years ago [1, 2], many problems with handling, materials, and contamination of these catheters have arisen. Catheterization of the femoral vessels produces more complications than the catheterization of the superior vena cava (SVC). Cannulation of the SVC versus the subclavian vein is difficult to implement and involves a higher complication rate [2, 3]. Using the infraclavicular catheterization technique, it is often difficult to push the large-bore catheter under the clavicle. Because of the anatomical position of the subclavian vein, perforation is more likely with a rigid, large-bore catheter, apart from the danger of causing a pneumothorax or a hemothorax [3–9].

Despite technical innovations in hemodialysis (HD) and apheresis, the problems of providing temporary or permanent vascular access appear to have found no satisfactory solution. Temporary vascular access, in particular, still presents considerable problems. Therefore, many investigators have inserted large-bore catheters in the superior vena cava rather than in the internal jugular or the subclavian veins [3–9]. Dialysis catheters are used for vascular access in 65% of incident hemodialysis patients, and in 25% of the prevalent HD populations [10]. Complication rates due to infections for venous catheters are reported to be between 34 and 40% and more [11, 12].

Synthetic catheter implants are increasingly used for intensive medical treatment and extracorporeal detoxifications procedures. Correspondingly, typical complications such as infections and thrombosis have also increased. Infections present a particular problem because they can appear at any time, even years after an implantation, and may affect all materials.

Catheter-related bacteremia is a major cause of morbidity among hemodialysis patients. Treatment with systemic antibiotics alone without removal of the catheter fails to definitively eradicate the infection in most patients [13]. Catheter-related bacteremia can be managed by either catheter removal with delayed placement of a new catheter or exchange of the infected catheter with a new catheter over a guide-wire and additional systemic antibiotic therapy.

The source of catheter-related bacteremia is in most patients a bacterial biofilm, which forms in the catheter lumen. This biofilm, mostly consisting of *Staphylococcus aureus*, cannot be destroyed or eliminated by a systemic antibiotic therapy because of antimicrobial resistance [14].

In 1981, Locci et al. demonstrated that bacterial could most of the time colonize artificial, rough surfaces [15]. The combination of tough surfaces and protein deposits should be an ideal situation for the colonization of bacteria. The bacteria could produce and become covered with a slime layer, in which case antibiotic drugs have no influence on the bacteria. The bacteria under the slime layer use the organic substances of the catheter material for their metabolism. The toxins of the bacteria can penetrate the slime layer and enter the patient blood provoking a catheter infection. Biofilm is a microbial-derived sessile community characterized by cells that are irreversibly attached to a substratum or to interface to each other, embedded in a matrix of extracellular polymeric substances that have produced [16]. Such a biofilm can be the origin of fibrin sheath formations leading to catheter dysfunction due to blood flow reducing and to blood disturbances. The therapy must be to remove the catheter immediately or exchange it over a guide-wire with a new catheter and additional systemic antibiotic therapy.

In addition to infection, biocompatibility of synthetic materials is a major problem. The interaction of blood with a synthetic surface causes coagulation and activation of the complement system. This can lead to the adsorption of various proteins and the formation of a layer of protein on the synthetic surface. Thrombocytes, other cells, and bacteria adhere to this layer of protein so that thrombi may form, which can lead to blood flow disturbances and catheter dysfunction [17]. Because of these problems, surface modification processes that can reduce the rate of infection or thrombogenicity, without adversely affecting basic catheter design and functionality, are of special interest.

The large-bore catheter has been frequently modified over recent years, and all models available are of similar construction with single, double, or triple lumen [18–25]. To influence catheter-related bacteremia, different new developments are available today, like coating of the catheter surface with antibiotic-heparin, cuffs on the outer surface, catheter for tunnelling, installation of an antibiotic-anticoagulant lock into the catheter lumen after the HD, and so forth, [13, 26, 27].

The authors introduced in 1979 the transcutaneous insertion of large-bore catheters through the internal jugular vein [11], and in 1992 they used for the first time available catheters which were coated on the outer surface with silver. In a retrospective study from 1992 to 2007, all catheters with surface treatment of silver versus untreated catheters were investigated after removal using a scanning electron microscope. Also, bacterial colonization and thrombus accumulation and the cuffs of the catheters after fixation were also investigated. In a preliminary study from 2001, the authors found a decline of the infection rate with the surface-treated catheter [28]. To examine these results in a 15-year study is the aim of this paper.

## 2. Catheter and Material

Most of the available single-, double- or triple-lumen catheters have some deficiencies depending on the material. Not all catheters are radiopaque. No problem is experienced with polyurethane catheters after the incorporation of contrast media; however, the latter material may affect catheter durability when using Teflon. This problem was overcome by making a thicker catheter wall, but this caused endothelial irritation and early thrombus formation. Catheters providing radio contrast are not absolutely necessary, however, because their position can be controlled more simply and gently with an intra-atrial electrocardiogram lead (ia ECG) [29]. The three most important criteria of any catheter material are a good tolerance, a low thrombogenicity, and a low infection rate [30, 31].

Rarely do the material properties perfectly match every requirement in a given application and biomaterials are no exception. For instance, although a candidate orthopedic material may have ideal mechanical properties, it may elicit a deleterious biological response, or a candidate biosensor with good electrical characteristics may corrode readily in the presence of body fluids [32]. Therefore, it often becomes necessary to strike a compromise so that a material has acceptable properties in each pertinent area. This compromise is often made between bulk and surface properties. For example, in a product such as a hemodialysis catheter, which demands both good flexibility and low surface friction, the best candidate may be a slippery, less flexible material rather than a more supple one with unacceptably high friction.

A wide spectrum of biomaterial surface properties, including biological, mechanical, chemical, and other properties that directly influence biocompatibility and functionality, can be modified. Surface engineering is generally considered when a “good” surface is not good enough, when devices would not function without it, or when product differentiation is desired [32].

The importance of surface-engineered biomaterials has been recognized by major medical device companies, because surface modification processes can reduce the rate of infection, thrombogenicity, and other catheter-related complications without adversely affecting the basic design function of catheters. 

Although the field is still essentially in its infancy, the range of services currently offered by surface treatment vendors is varied and continually expanding. Examples include conventional coating process such as depending and spraying, vacuum-deposition techniques (e.g., sputtering), and surface modification approaches such as diffusion (nitriding, carburizing), laser and plasma processes, chemical plating, grafting or bonding, and bombardment with energetic particles (as in plasma immersion or ion implantation). Of the available techniques, those based on ionised particle bombardment have been particularly successful in biomaterial surface modification, primarily because they combine versatility and low-temperature processing with superior process control, reliability, and reproducibility [32].

The ion-beam-based technology used for the treatment of catheters covered herein is ion-beam-assisted deposition (IBAD; Spi-Argent, Spire Corporation, Bedford, MA, USA) [33–35]. The process is typically performed at low temperature under high vacuum. The affected layer in the typical films deposited by the IBAD process is in the order of 1 *μ*m or less, so vacuum-compatible catheter materials may, therefore, be treated without adversely affecting bulk mechanical properties. The IBAD is line-of-sight process. This implies that only the outer surface of the catheters can be treated directly; however, parts with complicated geometries may be manipulated for uniform coverage of all surfaces. The ion-beam-assisted deposition of a silver coating was used [36].

Silver has been indicated as a good prospect for an infection-resistant coating material for catheters. The problem previously preventing the use of silver on catheters has been the inability to deposit adherent films of silver on flexible polymeric substrates. The IBAD process permits the formation of silver coatings at a relatively low temperature with extremely good adhesion that prevents delamination of the film during extended exposure to bodily fluids. The IBAD silver-deposited film has a low coefficient of friction, is highly uniform, and has demonstrated excellent adhesion. Biocompatibility testing consisted of a cytotoxicity test, and the USP Systemic Injection Test. Excellent results were obtained in both tests [32, 36–38].

## 3. Patients

The authors present the retrospective study from 1992 to 2007; the inclusion criteria were patients >18 years of age who requires a large-bore catheters (in-/outpatient), were free of bacteremia, and provided informed consent. The exclusion criteria were a pregnant or lactating female, a hypersensitivity of silver, and a bacteremia at the time of catheter insertion. An IRB approval was in 1992 not necessary.

In the study, a total of 159 patients (age 66.5 ± 13.2 years, females *n* = 94 (59%) are involved (Table 1)). Large-bore, single lumen catheters were inserted percutaneously in the internal jugular or subclavian veins. The percutaneously catheterisation was necessary in renal failure because of acute kidney injury (AKI) for hemodialysis due to cardiovascular disease, postoperative AKI, and so forth, and in end-stage renal disease (ESRD) because of clotting fistula, septicemia, abscess and catheter thrombosis, and faults in the catheter material (*n* = 138 (86.8%)) (Table 2). Further indications to catheterisation were access problems in patients with familial hypercholesterolemia (*n* = 12 (7.5%)), different indications for plasmapheresis (*n* = 7 (4.4%)), and in 2 patients with carcinoma (*n* = 2 (1.3%)).

In 54 patients (34%), a catheter with a silver coating on the outer surface (Spi-Argent, Spire, Bedford, MA, USA) was inserted, and 105 patients (66%) received untreated catheters. Patients with untreated catheters were younger (62.2 ± 16.2 versus 68.8 ± 10.7, *P* = 0.003), but there were no differences between the groups regarding gender distribution, diagnosis, or extracorporeal detoxification methods. The catheters were placed by nephrologists after the Seldinger technique and/or under fluoroscopic guidance. Before percutaneous insertion, each patient skin was disinfected using a consistent method, and a sterile skin smear was taken for microbiologic examination, and then the catheter was inserted. Before fixing the catheter with a suture, its position (particularly the catheter tip) should be checked with a normal radiological control and/or with an ia ECG [29]. In long-term catheters, a blood smear was taken every 4 weeks to screen for bacteria. Catheters were removed either when other vascular access routes became available or when serious infections developed, or if the catheter was not longer necessary.

Before catheter removal, a skin smear was taken. The catheters were then removed under sterile conditions, and the tip was examined bacteriologically. In the remainder, the catheter was rinsed in a physiological saline solution and fixed in a solution of phosphate buffer containing glutaraldehyde and formaldehyde for histological investigation.

## 4. Statistical Analysis

Statistical analysis was performed using the Statistical Package for Social Sciences (SPSS 13.0). All continuous data are presented as mean ± standard deviation (SD) or if the data showed no normal distribution, as median and range. Dichotomous data were presented as a number (*n*) or in percent (%). Univariate, unadjusted analyses were performed with the independent samples *t*-test, chi-square test, Fisher's exact test for frequencies at or below 5, and the Wilcoxon's rank sum test. Pearson's correlation coefficient was calculated and multivariate analysis was used to evaluate the presence of associated variables. Significance was defined at the 0.05 level.

## 5. Results

**Figure 1:**
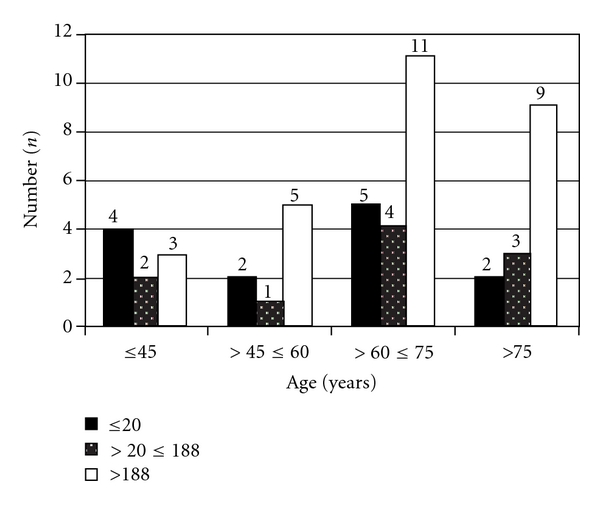
In situ times of treated and untreated catheters in patients after classification and age.

**Figure 2:**
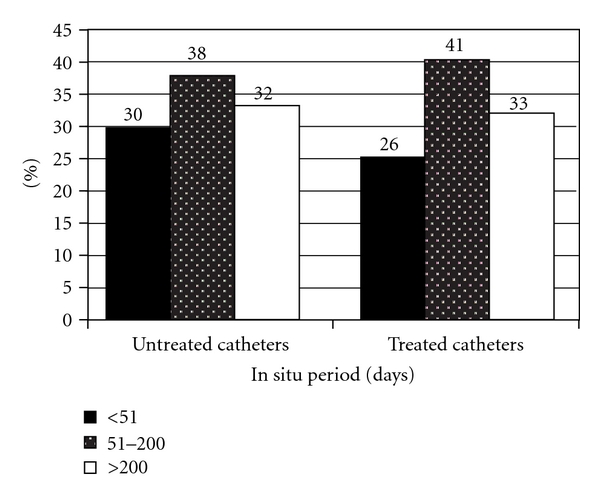
In situ periods of untreated and silver-coated catheters after classification for in situ times.

The median in situ period untreated and silver-coated catheters were 138.9 (range, 1–1,845) and 115.0 (range, 4–1,348) days, respectively, (*P* = 0.653). Calculating the in situ times after classification for different age groups, it will be overt, that in patients older than 45 years, in situ times were significantly longer (*P* < 0.01) (Figure 1). Comparing the in situ times of untreated catheters after classification for in situ times, there was a tendency towards longer in situ times for the silver-coated catheters (Figure 2). In the median, catheters were used for 44 (range, 1–670) treatment sessions. Untreated catheters were used for 51 (range, 1–625) treatments, silver-coated catheters for 39.0 (range, 1–670, *P* = 0.849) treatment sessions.

Performing microbiologic examinations, some differences were overt. Of the untreated catheters tips, 55% cultured positive for bacteria. Of the cultures in patients with surface-treated catheters, 52% were positive, not significantly lower. Although untreated catheters showed a lower infection rate with *Staphylococcus aureus*, in treated catheters the infection rate with *Staphylococcus epidermidis*, pseudomonas, and others such as saprophytes were not significantly lower (Table 3).

Performing multivariate analysis, there was a strong association between catheters' in situ period (*R*-square = 0.96), the number of treatment sessions (*β* = 0.97, *P* < 0.001), and patients' age (*β* = 0.095, *P* = 0.002). There was no association between the in situ time and silver-coated/untreated catheters, results of the bacteriological examination, and patients diagnosis or outcome. Catheter malfunction or fibrin sheath formation as an outcome of both groups was not investigated.

The decrease of the infection rate in surface-treated catheter in the preliminary study from 2001 cannot be seen in this presented study from 1992 to 2007. An explanation could be that all and more available data are now evaluated. The untreated catheters showed a higher positive culture for bacteria of 55% versus 52% to surface-treated catheters, but without significance. The procedure for both studies was the same.

## 6. Discussion

Catheter-related bacteremia and thrombosis are the most dangerous complications of large-bore catheter aside from accidental puncture of an artery. These catheter-related complications are contributing factors to increasing cost medical care. They are responsible for patient discomfort, morbidity, and occasional mortality. In addition to colonization, biocompatibility of a catheter material is an important contributing factor to a successful clinical outcome, particularly in catheters that remain in situ for several weeks or months. Though improved since the use of centrally placed catheters, the incidence of catheter clotting was previously very high.

Infection rates range from 5 to 30% and the most bacteria found is the *Staphylococcus aureus*. These rates do not depend on the route of vascular access [39, 40]. Catheter-related *Staphylococcus aureus* bacteremia is one of the main causes of morbidity and a preventable cause of death in hemodialysis. Patients on dialysis are at a high risk of *Staphylococcus aureus* bacteremia, and they have a four times higher mortality from central venous catheter-related *Staphylococcus aureus* bacteremia than other patients [14, 41, 42]. As such, new surgical techniques, catheter materials, and therapeutic drugs, and sterile handling during the treatments that influence performance and longevity of catheters are of great interest to the medical community [43, 44].

These catheter-related complications are contributing factors to the increasing cost of medical care. They are responsible for patient readmissions and longer hospital stays as well as patients discomfort, morbidity, and occasional mortality. Feldman et al. calculated in 1996, the costs of the morbidity due to catheter infections will soon exceed $1 billion per year [45]. Therefore, he demanded to reduce vascular-access-related morbidity and that strategies must be developed not only to prevent and detect appropriately early synthetic vascular access dysfunction, but to better identify the patients in a whom radial arteriovenous fistula is a viable clinical option.  Table 4 shows representative health care cost savings for hemodialysis catheters, given specific infection rates and potential infection rate reductions achieved by treated catheters [35]. The cost analysis was calculated using the literature and the available costs of different companies, which distribute these catheters [46].

To reduce infection rates and thrombogenicity, coated catheters and cuffs were investigated [47–52]. The clinical results of our preliminary investigations showed a significantly reduced infection rate in treated versus untreated catheters, a reduction of more than 75% [28]. With the silver surface treatment, a very smooth metallic surface was obtained, which was responsible for a lower thrombogenicity rate. The activation of coagulation factors at the catheter surfaces was not investigated. Silver ions are bactericidal, therefore, no bacteria growth is possible on the treated catheter surface. The positive association between the in situ time of the catheters and the patients' age maybe because of an alteration of the immune system in elderly patients, especially in hemodialysis patients.

But in our retrospective study of all silver-coated catheters no significant reduction in infection rate, improvement, or life expectancy of silver-coated versus untreated catheters, which were inserted during 1992–2007, was observed. One reason can be that with the IBAD technology, only the outer surface is coated with silver. The postulated penetration of silver ions from the outer to the inner surface cannot be shown with these results. The only outer-surface-treated catheters with silver have no advantage in point of view of reducing infection rate and improvement of patients versus the untreated catheters. The handling of the catheters before, during, and after the extracorporeal treatments cannot prevent the contamination with bacteria, especially the untreated inner side.

Based on these results, new materials must be developed, which should have better biocompatibility to reduce side effects so that they can be left in situ for a long time, because the part of dialysis in patients with vascular problems is increasing in the last decade, because the age of HD patients is permanently growing up. As the requirement for more and more artificial organs and/or organ replacements increases, especially in elderly patients, there will be a definite need for new materials with better biocompatibility and for suitable technologies to solve these infection, thrombosis, and medical problems to reduce the costs and get a better improvement of patients.

This requirement shows perhaps the new developed catheter material, the microdomain-structured surface (PUR-SMA-coated catheters, Gambro, Germany) [28]. Microdomain surfaces are considered the most biocompatible because they mimic the structure of natural biological surfaces. Microdomain structures are used to match the multiple requirements for improved catheter surfaces, that is reduced thrombogenicity and improved antimicrobial properties. An SMA-modified polyurethane coating consists of hydrophobic and hydrophilic microdomains in range below 50 nm. Up to 50 percent of the SMA molecule is presented to the surface and creates microdomain structures surfaces. If the domains are below a critical dimension of approximately 100 nmm, theoretical considerations indicate that interaction with proteins, blood cells, or even bacteria will be unstable and therefore, not occur as frequently as on non-microdomain structured surfaces.

The new PUR-SMA coating prevents contact of blood components with barium sulfate, possibly leading to leaching as particles or dissolving in the surrounding media. The advantage of the PUR-SMA surface treatment is the coating of the inner and the outer surface in contrast to the ion-beam-based surface treatment technologies in which can be treated only the outer surface of the catheters. The preliminary results with these PUR-SMA-coated catheters showed a good biocompatibility without any blood deposits and a low thrombogenicity and coagulation activity. The microbiological results were low and of those from the Spi-Argent catheters [53].

More new materials must be developed, which should have better biocompatibility to reduce side effects so that they can be left in situ for a long time, because the part of dialysis in patients with vascular problems is increasing in the last decade. As the requirement for more and more artificial organs and/or organ replacements increases, there will be a definite need for new materials with better biocompatibility and for suitable technologies to solve these infection, thrombosis, and medical problems to reduce the costs and get a better improvement of patients. But it appears impossible to create a surface with an absolute “zero” adherence due to thermodynamical reasons and due to the fact that a modified material surface is in vivo rapidly covered by plasma and connective tissue proteins.

Therefore, other concepts of the prevention of implant-associated infections must involve the impregnation of the devices in the inner and outer surface with antibiotics, antimicrobial substances, and/or metals [54]. Another point is to understand the processes leading to the development of catheter-related bacteremia in order to offer effective preventative and therapeutic possibilities [55].

## 7. Conclusion

In a retrospective study from 1992 to 2007, outer-surface-treated catheters with silver versus untreated catheters in 159 patients, who needed a large-bore catheter, were investigated. The results of a preliminary study from 2001, which showed 75% decline in the infection rate with the surface-treated catheters, cannot be confirmed with the presented study. There was no association between the in situ time and silver-coated/uncoated catheters, results of the bacteriological examination, and patients diagnosis or outcome. One reason maybe that in the surface-treated catheters only the outer surface was coated with silver and another reason is the possibility of contamination by the handling during the extracorporeal treatments. Therefore, new materials and surface treatment technologies are needed to save health care costs for hemoldiaysis catheters, to reduce infection rates and thrombus formations and help to improve the patients outcome.

## Figures and Tables

**Table 1:** Characteristics of 159 patients who received large-bore catheters for dialysis or apheresis.

Parameter	Mean ± SD
Age (30–82 years)	66.5 ± 13.2
Females (*n* (%))	94 (59%)
Treated surface catheters (silver) (*n* (%))	54 (34%)
In situ time (days)	217.6 ± 285.8 (Median 123.0, 1–1845)
Treatments (*n*) (dialysis, apheresis)	76.4 ± 103.4 (Median 44.0, 1–670)

**Table 2:** Indications for the insertion of large-bore catheters (*n* = 159).

Indications	(*n*)	%
Renal failure		
Acute kidney injury (AKI)	40	25.2
Clotting fistula	34	21.4
Septicemia (catheter-related)	29	18.2
Abscess (catheter-related)	8	5.0
Bleeding (catheter-related)	4	2.5
Catheter thrombosis and faults in catheter material	23	14.5
Hypercholesterolemia		
LDL-apheresis	10	6.3
Septicemia	2	1.24
Plasmapheresis		
Different indications	6	3.8
Removal by patient	1	0.62
Carcinoma		
Removal by patient	2	1.24

**Table 3:** Microbiological examinations of 105 untreated and 54 surface-treated catheters.

Microorganisms	Untreated (*n*)	%	Treated (*n*)	%	*P* value
Negative	47	45	26	48	n.s.
*S. aureus*	31	29	21	38	n.s.
*S. epidermidis*	7	7	1	2	n.s.
Pseudomonas	1	1	0	0	n.s.
Enterobacter	1	1	1	2	n.s.
Others	18	17	5	10	n.s.

**Table 4:** Potential health care cost reductions that could be achieved through the use of surface-treated catheters [45].

Device	Hemodialysis	Average infection (%)
Annual usage (devices)	125,971	
Infection rate (%)	5–20	Rate: 12
Cost ($) of complication (due to infection)	3.517	
Cost ($) of coating	12	
Reduction of infections (%)	10–65	Reduction 40
Market size (1997) ($)	12.6 million	
Price ($) of each device (surface treatment)	120	
Savings ($) per year by using surface-treated devices	17.7 milion	Reduction 40
